# Supplementary material for: The Risk of Malnutrition and Sarcopenia in Elderly People Living with HIV during the COVID-19 Pandemic
Source: Nutrients. 2024 Aug 2;16(15):2540. doi: 10.3390/nu16152540 (PMC11313797; doi:10.3390/nu16152540)
Supplement: Supplementary file 1 [file nutrients-16-02540-s001.zip › nutrients-3114849-supplementary.pdf]

Table S1. Responses to each question of the Mini Nutritional Assessment by nutritional status.

	Item	Total (N = 177)		Normal Nutritional Status (N = 127)		Abnormal Nutritional Status (N = 50)		*p*-Value *
		N	%	N	%	N	%	
A	Has food intake declined over the past 3 months due to loss of appetite, digestive problem, chewing, or swallowing difficulties?							<0.001
	0 = severe decrease in food intake	3	1.7	0	0	3	6	
	1 = moderate decrease in food intake	42	23.7	17	13.4	25	50	
	2 = no decrease in food intake	132	74.6	110	86.6	22	44	
B	Weight loss during the last 3 months							<0.001
	0 = weight loss greater than 3 kg (6.6lbs)	7	4	2	1.6	5	10	
	1 = does not know	7	4	2	1.6	5	10	
	2= weight loss between 1 and 3 kg (2.2 and 6.6lbs)	49	27.7	30	23.6	19	38	
	3= no weight loss	114	64.3	93	73.2	21	42	
C	Mobility							0.001
	0 = bed or chair bound	3	1.7	0	0	3	6	
	1 = able to get out of bed/chair but does not go out	2	1.1	0	0	2	4	
	2 = goes out	172	97.2	127	100	45	90	
D	Has suffered psychological stress or acute disease in the past 3 months?							<0.001
	0 = yes	16	9	4	3.1	12	24	
	2 = no	161	91	123	96.9	38	76	
E	Neuropsychological problems							<0.001
	0 = severe dementia or depression	1	0.6	0	0	1	2	
	1 = mild dementia	31	17.5	12	9.4	19	38	
	2 = no psychological problems	145	81.9	115	90.6	30	60	
F	Body mass index (BMI) = weight in kg/ (height in m)^2							<0.001
	0 = BMI less than 19	16	9	3	2.4	13	26	
	1 = BMI 19 to less than 21	31	17.5	16	12.6	15	30	
	2 = BMI 21 to less than 23	32	18.1	25	19.6	7	14	
	3 = BMI 23 or greater	98	55.4	83	65.4	15	30	

G	Lives independently (not in nursing home or hospital)							0.578
	1 = yes	173	97.7	123	96.9	50	100	
	0 = no	4	2.3	4	3.1	0	0	
H	Takes more than 3 prescription drugs per day							0.020
	0 = yes	122	68.9	81	63.8	41	82	
	1 = no	55	31.1	46	36.2	9	18	
I	Pressure sores or skin ulcers							0.297
	0 = yes	11	6.2	6	4.7	5	10	
	1 = no	166	93.8	121	95.3	45	90	
J	How many full meals does the patient eat daily?							<0.001
	0 = 1 meal	0	0	0	0	0	0	
	1 = 2 meals	43	24.3	19	15	24	48	
	2 = 3 meals	134	75.7	108	85	26	52	
K	Selected consumption markers for protein intake							
	At least one serving of dairy products (milk, cheese, yoghurt) per day	89	50.3	67	52.8	22	44	0.294
	Two or more servings of legumes or eggs per week	164	92.7	117	92.1	47	94	0.667
	Meat, fish or poultry everyday	149	84.2	110	86.6	39	78	0.157
	0.0 = if 0 or 1 yes	25	14.1	17	13.4	8	16	0.212
	0.5 = if 2 yes	77	43.5	51	40.2	26	52	
	1.0 = if 3 yes	75	42.4	59	46.4	16	32	
L	Consumes two or more servings of fruits or vegetables per day?							0.003
	0 = no	27	15.3	13	10.2	14	28	
	1 = yes	150	84.7	114	89.8	36	72	
M	How much fluid (water, juice, coffee, tea, milk…) is consumed per day?							0.013
	0.0 = less than 3 cups	50	28.2	28	22	22	44	
	0.5 = 3 to 5 cups	29	16.4	22	17.3	7	14	
	1.0 = more than 5 cups	98	55.4	77	60.7	21	42	
N	Mode of feeding							NA
	0 = unable to eat without assistance	0	0	0	0	0	0	
	1 = self-fed with some difficulty	0	0	0	0	0	0	
	2 = self-fed without any problem	177	100	127	0	50	0	
O	Self-view of nutritional status							<0.001

	0 = views self as being malnourished	4	2.3	1	0.8	3	6	
	1 = is uncertain of nutritional state	44	24.9	21	16.5	23	46	
	2 = views self as having no nutritional problem	129	72.8	105	82.7	24	48	
P	In comparison with other people of the same age, how does the patient consider his/her health status?							<0.001
	0.0 = not as good	7	4	2	1.6	5	10	
	0.5 = does not know	17	9.6	7	5.5	10	20	
	1.0 = as good	73	41.2	48	37.8	25	50	
	2.0 = better	80	45.2	70	55.1	10	20	
Q	Mid-arm circumference (MAC) in cm							<0.001
	0.0 = MAC less than 21	2	1.1	0	0	2	4	
	0.5 = MAC 21 to 22	13	7.4	4	3.1	9	18	
	1.0 = MAC greater than 22	162	91.5	123	96.9	39	78	
R	Calf circumference (CC) in cm							<0.001
	0 = CC less than 31	30	16.9	10	7.9	20	40	
	1 = CC 31 or greater	147	83.1	117	92.1	30	60	

* The chi-squared test or Fisher's exact test was used, as appropriate
